# The Performance of Pulmonary Function Tests in Predicting Systemic Sclerosis—Interstitial Lung Disease in the European Scleroderma Trial and Research Database

**DOI:** 10.3390/diagnostics14030295

**Published:** 2024-01-30

**Authors:** Gemma Lepri, Cosimo Bruni, Lorenzo Tofani, Alberto Moggi-Pignone, Martina Orlandi, Sara Tomassetti, Michael Hughes, Francesco Del Galdo, Rosaria Irace, Oliver Distler, Valeria Riccieri, Yannick Allanore, Ana Maria Gheorghiu, Elise Siegert, Jeska De Vries-Bouwstra, Eric Hachulla, Mohammed Tikly, Nemanja Damjanov, Francois Spertini, Luc Mouthon, Anna-Maria Hoffmann-Vold, Armando Gabrielli, Serena Guiducci, Marco Matucci-Cerinic, Daniel Furst, Silvia Bellando-Randone, EUSTAR Collaborators

**Affiliations:** 1 Division of Rheumatology, AOU Careggi, University of Florence, 50121 Florence, Italy; 2 Department of Rheumatology, University Hospital Zurich, University of Zurich, 8006 Zurich, Switzerland; 3 Division of Internal Medicine, AOU Careggi, University of Florence, 50121 Florence, Italy; 4 Interventional Pulmonology Unit, AOU Careggi, University of Florence, 50121 Florence, Italy; 5 Department of Rheumatology, Sheffield Teaching Hospitals NHS Foundation Trust, Royal Hallamshire Hospital, Sheffield S10 2JF, UK; 6 Raynaud’s and Scleroderma Programme, NIHR Biomedical Research Centre and Leeds Institute of Rheumatic and Musculoskeletal Medicine, University of Leeds, Leeds LS2 9JT, UK; 7 Rheumatology Unit, Department of Precision Medicine, University of Campania “Luigi Vanvitelli”, 81100 Naples, Italy; 8 Rheumatology Unit, “Sapienza” University, 00185 Rome, Italy; 9 Rheumatology Department, Hopital Cochin, University of Paris, 75019 Paris, France; 10 Internal Medicine & Rheumatology Department, Cantacuzino Hospital, Carol Davila University of Medicine and Pharmacy, 050474 Bucharest, Romania; 11 Rheumatology, Charite University Hospital, 10117 Berlin, Germany; 12 Department of Rheumatology, Leiden University Medical Center, 2333 ZA Leiden, The Netherlands; 13 Service de Médecine Interne, Centre Hospitalier Universitaire, 59000 Lille, France; 14 Department of Internal Medicine, Division of Rheumatology, Chris Hani Baragwanath Academic Hospital, University of the Witwatersrand, Johannesburg 1864, South Africa; 15 Institute of Rheumatology, University Belgrade Medical School, 11000 Belgrade, Serbia; 16 Service Immunologie et Allergie, CHUV, 1005 Lausanne, Switzerland; 17 National Referral Center for Rare Systemic Autoimmune Diseases, Hôpital Cochin, University Paris Descartes, 75006 Paris, France; 18 Department of Rheumatology, Oslo University Hospital, 0372 Oslo, Norway; 19 Department of Clinical and Molecular Science, Università Politecninca delle Marche, 60121 Ancona, Italy; 20 Unit of Immunology, Rheumatology, Allergy and Rare Diseases (UnIRAR), IRCCS San Raffaele Hospital, 20132 Milan, Italy; 21 Division of Rheumatology, University California Los Angeles, Los Angeles, CA 90095, USA

**Keywords:** computed tomography (CT), interstitial lung disease, scleroderma, pulmonary function test

## Abstract

Background and Objectives: In SSc, ILD is a major cause of morbidity and mortality. We aimed to investigate the performance of DLCO (diffusing capacity of lung carbon monoxide) and FVC (forced vital capacity) delta change (Δ) and baseline values in predicting the development of SSc-ILD. Methods: Longitudinal data of DLCO, FVC, and ILD on the HRCT of SSc patients from the EUSTAR database were evaluated at baseline (t_0_) and after 12 (±4) (t_1_) and 24 (±4) (t_2_) months. Results: 474/17805 patients were eligible for the study (403 females); 46 (9.7%) developed ILD at t_2_. Positivity for anti-topoisomerase antibodies (117 patients) showed an association with ILD development at t_2_ (*p* = 0.0031). Neither the mean t_0_ to t_1_ change (Δ) of DLCO nor the mean t_0_ to t_1_ FVCΔ predicted the appearance of ILD at t_2_. Investigating the possible role of baseline DLCO and FVC values in predicting ILD appearance after 24 (±4) months, we observed a moderate predictive capability of t_0_ DLCO < 80%, stronger than that of FVC < 80%. Conclusions: We suggest that an impaired baseline DLCO may be predictive of the appearance of ILD after 2 years of follow-up. This result advances the hypothesis that a reduction in gas exchange may be considered an early sign of lung involvement. However, further rigorous studies are warranted to understand the predictive role of DLCO evaluation in the course of SSc.

## 1. Introduction

Systemic sclerosis (SSc) is characterized by small-vessel vasculopathy, immune dysregulation, and fibroblast dysfunction leading to the fibrosis of skin and internal organs [1]. Interstitial lung disease (ILD), together with pulmonary arterial hypertension (PAH), represents the main cause of SSc-related deaths [2,3,4], and it occurs more frequently in the diffuse cutaneous subset (dcSSc) [5]. Despite the recent advances in SSc-ILD treatment, lung involvement represents a fearsome manifestation of the disease, and the optimal timing to start an immunosuppressive and antifibrotic treatment still remains debated [6]. Recent studies also highlighted a certain involvement of a small airway in SSc patients, which may be associated with ILD [7]. A study on a Norwegian SSc cohort reported an ILD prevalence on HRCT (high-resolution computed tomography) of 50% [8]. The clinical course and the timing of onset of ILD are unpredictable among SSc patients, and ILD may occur in the early stages, particularly in dcSSc patients [2,9,10,11]. Symptoms due to lung involvement, such as dyspnoea and cough, can be delayed and are not specific to ILD. Therefore, an early diagnosis, a regular follow-up [12], and the identification of predictors of evolution are crucial and eagerly awaited to improve SSc-ILD management. In this context, besides HRCT which represents the mainstay for ILD diagnosis, other non-ionizing methods of lung evaluation such as lung ultrasound are under study [13].

The evidence-based expert European consensus confirmed HRCT as the primary tool for the screening and diagnosis of SSc-ILD, with pulmonary function tests (PFTs) and clinical symptoms providing supporting evidence [14]. The baseline forced vital capacity (FVC) value has been proposed as a reliable predictor of SSc pulmonary function deterioration [15,16,17]. In addition, the recent functional criteria for progressive pulmonary fibrosis reported an absolute decline in FVC of ≥5% or in diffusion of the lung for carbon oxide (DLCO) ≥10% within 1 year of follow-up as physiological evidence of disease progression, when not explained by other causes [18]. In SSc, a change in DLCO or in DLCO per unit alveolar volume (DLCO/AV) change ≥15% has been proposed as a marker of poor ILD prognosis in patients with signs of ILD at enrolment in clinical trials [19]. In addition, the combination of lung fibrotic involvement greater than 30% on HRCT or between 10 and 30% fibrotic extent with a predicted FVC <70% are associated with premature mortality [20]. Indeed, in patients with extensive disease, a decline in FVC ≥10% or a decline in FVC of 5–9% together with a decline in DLCO > 15% predicted mortality over a 15-year follow-up period [21]. Despite the confirmed important and complementary role of HRCT and PFTs in SSc-ILD diagnosis and management, there has been controversy regarding the usefulness of the DLCO and FVC as predictors of ILD onset [22,23,24]. In fact, although PFTs are recommended for the early detection of ILD progression, they seem inadequate as the only screening tool for the diagnosis of ILD [25,26].

The aim of our study was to investigate the role of DLCO and FVC in predicting the development of ILD by HRCT after a follow-up of 2 years, using the prospectively collected, longitudinal European Scleroderma Trial and Research (EUSTAR) database. The primary endpoint of this study was to evaluate the efficiency of the DLCO (DLCOΔ) and FVC (FVCΔ) delta change over 12 months from baseline to predict the subsequent ILD onset at 24 months. As secondary endpoints, the study evaluated and compared the clinical characteristics of patients with and without ILD at the end of observation, including the predictive capability of baseline DLCO and FVC values.

## 2. Patients and Methods

We evaluated SSc patients from the EUSTAR database who satisfied the 1980 American Rheumatology Association (ARA) and/or the 2013 American College of Rheumatology/European League Against Rheumatism (ACR/EULAR) classification criteria for SSc [27,28]. Each EUSTAR centre received approval by the local ethics committee, and written informed consent was locally acquired for registered patients. Patients with available longitudinal data of DLCO, FVC, and HRCT at baseline (t_0_) and after 12 (±4) (t_1_) and 24 (±4) (t_2_) months were eligible for the study, but only patients with a negative HRCT at t0 and t_1_ were included.

HRCT was defined as negative when no signs of ILD were evident according to the local radiology evaluation, as entered in the database. Exclusion criteria included the following: an absence of PFTs and HRCT at t_0_, t_1_, and t_2_, juvenile disease onset, age < 18 years, the presence of ILD on HRCT at t_0_ or t_1_, a lack of follow-up visit, and a diagnosis of PH anytime during the study period (as entered in the database or a value of mPAP ≥ 25 mmHg).

The DLCOΔ (change from t_0_ to t_1_) and FVCΔ (change from t_0_ to t_1_) for the prediction of ILD appearance at t_2_ were calculated. We considered DLCO > 80% and FVC > 80% as normal values [29].

### Statistical Analysis

Mean ± SDs were reported for continuous variables, while absolute and relative frequencies for each category were reported for categorical or dichotomous variables.

To evaluate differences between continuous variables, Student’s *t*-test, *t*-test with Satterthwaite adaptation, or Mann–Whitney Test were used, according to the results of Shapiro–Wilk’s test for normality distribution and Bartlett’s test for homoskedasticity.

To evaluate association between categorical variables, the chi-square test or Fisher exact test were used according to the frequencies in table cells. To assess the association between binary outcomes and risk factors, a logistic regression model was used. For each risk factor OR, 95% confidence intervals (CI95%) and *p*-values were reported. In addition, the predictive capability of each risk factor was represented by area under the ROC curve (AUROC) and its CI95% (considering the predictive capability to be scarce for an AUROC of 0.5–0.6; moderate for 0.6–0.7; good for 0.7–0.8; high for 0.8–0.9; and excellent for 0.9–1).

## 3. Results

### 3.1. Study Population

**Figure 1:**
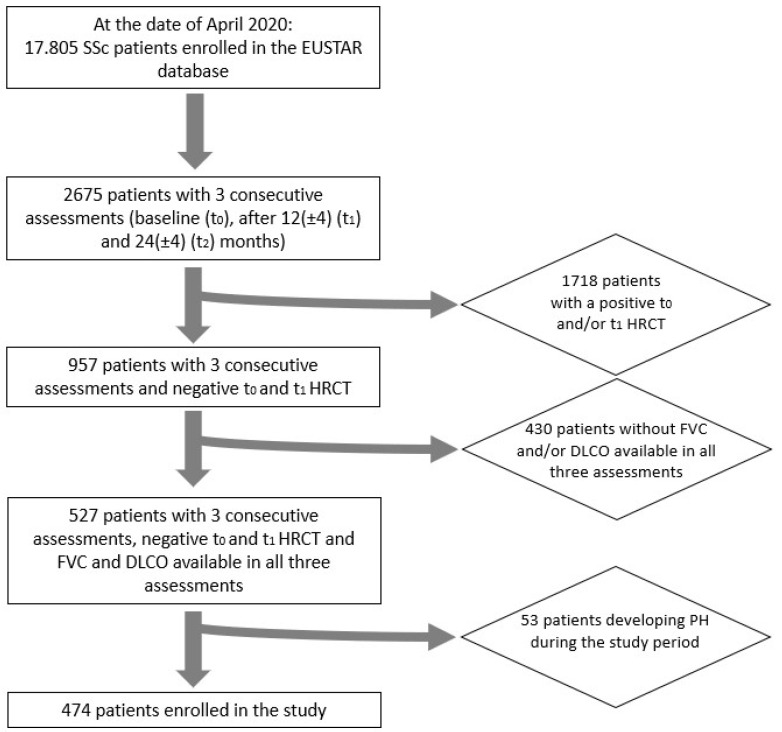
Flowchart of patient enrolment.

On 30 April 2020, among 17.805 SSc patients in the EUSTAR database, 527 patients had complete longitudinal data on PFTs and HRCT at t_0_, t_1_, and t_2_. Out of 527 patients, 53 were excluded for developing PH during the observation period, leading to the analysis of 474 patients from 39 EUSTAR centres (Figure 1). As expected, we noted a high female predominance (403 females, 85%), 220 (48.0%) patients were anticentromere (ACA)-positive, and 117 (25.4%) anti-topoisomerase I (Topo-I)-positive. Among the skin disease subsets, the limited cutaneous one (lcScc) was the most frequent (58.3%) followed by the dcSSc (26%), and 11.9% of patients presented only sclerodactyly. Regarding the capillaroscopic evaluation, the active scleroderma pattern was the most frequent (44.6%). The demographic and clinical characteristics are summarized in Table 1.

Among the enrolled population, 46 (9.7%) developed HRCT signs of ILD at t_2_.

The comparison of patients with and without ILD at t_2_ showed some different features at baseline (Table 2). Positivity for Topo-I antibodies was associated with ILD development (16.7% vs. 7.8%, *p* = 0.0031), contrarily to the positivity for ACA antibodies, which was negatively associated (4.4% vs. 14.4%, *p* = 0.0001). The disease duration and the extent of skin involvement were different in the two groups, without reaching statistically significant values (Table 2). In our population, the progression of skin involvement assessed by the modified Rodnan skin score (mRSS) from t_0_ to t_1_ did not correlate with ILD appearance [(OR (IC): 0.713 (0.35–1.46), *p* = 0.3521)].

### 3.2. Pulmonary Function Trends

The mean value of DLCO and FVC as a percent (%) predicted at t_0_, t_1_, and t_2_ in patients with positive and negative HRCT for lung involvement at t_2_ are shown in Table 2. Interestingly, it must be pointed out that patients with a positive HRCT at t_2_ also had significantly lower values of DLCO and FVC at t_0_ and t_1_ in comparison to patients with a negative HRCT at t_2_ (Table 2).

### 3.3. Primary End Point

We found no statistically significant difference in the mean DLCOΔ and FVCΔ from t_0_ to t_1_ in the two populations (negative vs. positive t_2_ HRCT). The mean DLCOΔ in patients with negative t_2_ HRCT and positive t_2_ HRCT was −0.5 (±12.6) and −1.0 (±15.1), respectively. The mean FVCΔ in patients with negative t_2_ HRTC and positive t_2_ HRCT was −0.2 (±10.6) and 0.1 (±11.5), respectively. None of them predicted the appearance of ILD at t_2_ [DLCOΔ: OR 0.997 (95%CI 0.97–1.02), *p* = 0.8024; FVCΔ: OR 1.002 (95%CI 0.97–1.03), *p* = 0.8664].

### 3.4. Secondary End Point

The enrolled population was divided into four groups according to DLCO trends from t_0_ to t_1_ (normal cut-off of DLCO ≥ 80%):

- 161 patients maintained a normal DLCO;
- 47 moved from a normal to a reduced DLCO;
- 38 patients presented a reduced DLCO at t_0_ and recovered beyond normality at t_1_;
- 228 patients had a reduced DLCO both at t_0_ and at t_1_.

A higher percentage of patients with ILD at t_2_ was found in groups III (5, 13.3%) and IV (31, 13.3%) [OR 2.56 (95%CI 0.81–8.13), *p* = 0.1112 and OR: 2.66 (95%CI 1.23–5.75), *p* = 0.0130, respectively]. Therefore, it should be noted that among patients with a positive t_2_ HRCT, the majority had a reduced DLCO at baseline (Table 3).

The same analysis was performed for FVC% predicted (the normal cut-off of FVC% predicted is ≥80%), identifying four similar subgroups:

- 415 patients maintained a normal FVC;
- 16 patients moved from a normal to a reduced FVC;
- 11 patients presented a reduced FVC at t_0_ and recovered beyond a normality of ≥80% at t_1_;
- 32 patients had a reduced FVC both at t_0_ and at t_1_.

Considering FVC trend subgroups, the higher percentages of patients with a positive HRCT at t_2_ were in groups II (4, 25.5%) and IV (6, 18.9%) [OR 3.62 (95%CI 1.11–11.82), *p* = 0.0331 and OR 2.51 (95%CI 0.97–6.50), *p* = 0.0588, respectively]. Among patients with ILD at t_2_, the majority had a normal FVC both at t_0_ and at t_1_ (Table 3).

**Figure 2:**
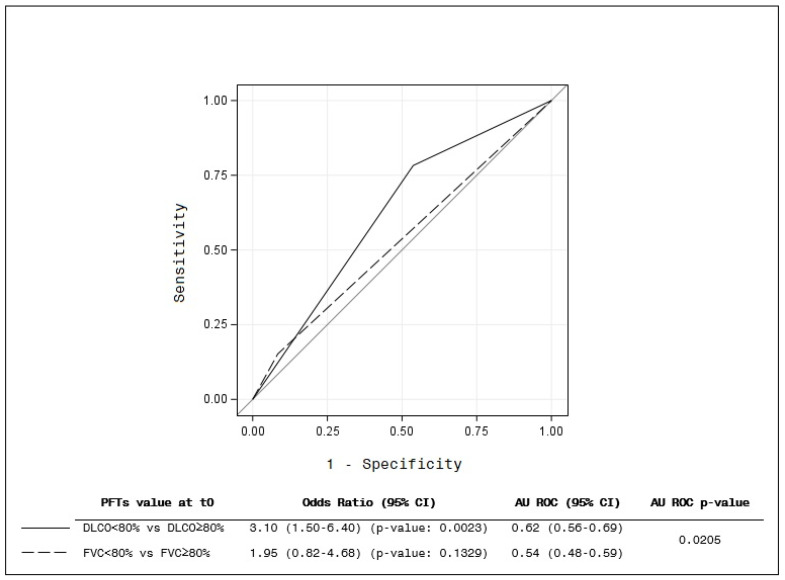
Prediction of t_0_ value of DLCO and FVC. The figure shows that an impaired baseline DLCO value (<80%) had more predictive capability of a positive HRCT after 2 years of follow-up than an abnormal baseline FVC (<80%) (*p* = 0.0205).

The most interesting result is that, at baseline (t_0_), the distinction between DLCO < 80% and DLCO ≥ 80% showed the best prediction for ILD development at t_2_ HRCT (AUROC IC95%) (*p* = 0.0205) (Figure 2). The figure shows that an impaired baseline DLCO value (<80%) had more predictive capability of a positive HRCT after 2 years of follow-up than an abnormal baseline FVC (<80%).

## 4. Discussion

In SSc, the prevalence of ILD ranges from 47 to 84% and it is associated with significant mortality and morbidity [3,30,31,32]. It is now well known that SSc-lung involvement may be progressive or remain stable for longer periods [33]. The heterogeneity of ILD evolution is still a challenge, and it is therefore of primary importance to identify the best tool and the correct timing to detect and predict lung involvement.

Many efforts have been made to identify the predictors of ILD onset and in this context; the main result of our study suggests a certain role of baseline DLCO in its prediction. In fact, it is pivotal to identify ILD predictors, and this might help to follow-up SSc patients, identifying those at higher risk of developing ILD [34,35]. Some investigations focused on the possibility to predict the progression of lung involvement in SSc-ILD patients. Among these, the SPAR (SPo2 ARthritis) model predicted ILD progression, helping to detect patients at risk for progressive fibrosis with the combination of lower peripheral capillary oxygen saturation (after a 6 min walking test) and arthritis [36]. Ahmed et al. reported an association between baseline FVC and DLCO values and survival in SSc-ILD patients, identifying a threshold of 70% FVC% predicted and 77% DLCO% predicted [37]. In SSc-ILD patients, Volkmann et al. found that the decline in FVC and DLCO over 2 years was a better predictor of mortality compared to baseline FVC and DLCO values [38]. Morisset J et al. suggested the SADL (smoking history, age, and DLCO% predicted) model as a mortality risk predictor for SSc-ILD patients [30]. Clearly, these data highlight the importance of PFTs values in the prediction of ILD prognosis.

The present study was focused on predicting the appearance of SSc-ILD on HRCT by using PFT parameters. The primary endpoint evaluating DLCOΔ and FVCΔ between t_0_ and t_1_ as a predictor of ILD onset at t_2_ HRCT was not met, and this might be related to the mean disease duration of the study population (about 6 years at t_0_), too long to use the Δ change to predict ILD. However, the secondary endpoint analysis suggested that even when signs of ILD are not yet present on HRCT, patients with a low baseline DLCO may have an increased risk of developing ILD after 2 years of follow-up.

Our population presented mild lung involvement showing an average non-significant functional decline in DLCO and FVC at one year of follow-up, and this probably influenced the success of the primary endpoint analysis. To evaluate the predicting role of DLCOΔ and FVCΔ, a larger population with a follow-up longer than two years would probably be required. On the other hand, the straightforward use of the baseline mean values of DLCO and FVC showed that the DLCO was better than the FVC for predicting a new ILD after 2 years. Dichotomizing these values (with abnormal defined as <80% for both DLCO and FVC), “abnormal” DLCO patients had a higher risk than “abnormal” FVC patients of developing HRCT-ILD after 2 years of follow-up. Into clinical practice, this datum may indicate the need to follow-up with HRCT SSc patients with a baseline impaired DLCO for at least 2 years. However, considering the “within-patient” variability in PFTs, perhaps it would be better to rely on more than a single measure to predict the development of ILD. Unfortunately, the design of our study ruled out all patients with ILD at HRCT at t_1_, preventing the possibility to evaluate the risk of developing ILD in a shorter follow-up.

At t_2_, our data showed normal mean FVC values in patients with ILD; however, despite the normal mean value, patients with ILD had a lower FVC than those without this complication. Considering DLCO, our data showed that in patients with ILD, the mean DLCO value over the study period was significantly lower than in patients with negative HRCT at t_2_. It is remarkable that patients without signs of ILD at t_2_ showed a DLCO near the normal cut-off (80%) at all the three assessments. This evidence may suggest an early impairment of the alveolar epithelium, probably confirming inflammatory involvement of the alveolar membrane as the primum movens in SSc-ILD pathogenesis [39]. Our data are in line with Tashkin et al., who found that DLCO provides the best overall estimate of HRCT-measured lung disease in SSc patients [34].

Our study confirmed some previous evidence, indicating a higher prevalence of Topo-I antibodies and the dcSSc subset among patients who developed ILD during the follow-up. In line with our results, previous data reported the association between dcSSc, African American ethnicity, shorter disease duration, and an older age at disease onset and the risk of developing SSc-ILD [40,41].

An association between progressive skin fibrosis within one year and a decline in lung function has been reported via analysing dcSSc patients from the EUSTAR cohort [42]. In our study, the change in mRSS seemed not to predict ILD onset. However, our population was probably too heterogeneous and with mild skin involvement, mostly presenting with only sclerodactyly or a limited subset.

Our study presents some limitations, mostly related to the data collected in the EUSTAR database. Data regarding the extent of ILD and qualitative data about pulmonary involvement in patients with ILD at t_2_ were mostly missing (no available data about reticulation changes, traction, and/or honeycombing changes; information about ground-glass opacities was only recorded in about 37%). PFTs were performed in 39 different centres, between 2003 and 2019, making the percent predicted less uniform and more variable. In addition, absolute values were not recorded, the haemoglobin value was not available for all patients, and the sample size was reduced when patients were divided into groups according to the trends in DLCO and FVC over time. In fact, some patients might have a negative baseline HRCT with a lower PFTs value, probably due to an incorrect execution of PFTs, as some of these patients presented a normal value of FVC at t_1_. In addition, the absence of a centralized reading of the HRCT scans or the presence of different HRCT acquisition protocols, as well as the absence of data on smoking (or other environmental factors) for all patients, must be listed. It is important to note that, in our study, no other correlations between ILD and biological data, other than autoantibodies, were determined as, unfortunately, some biological features were missing for enrolled patients. Beyond these weaknesses, the strengths of our study are represented by the number of enrolled patients (474 patients) and the long follow-up, as the study focused on PFTs compared to HRCT for at least 48 months in all patients, suggesting a predictive value of baseline DLCO.

Predictors of ILD onset are eagerly awaited to improve SSc-ILD management. Our study confirms the known risk factors in the identification of an SSc population at higher risk of developing ILD, including Topo-I positivity, the dcSSc subset, and a shorter disease duration from the appearance of Raynaud’s phenomenon. Our data suggest that the baseline DLCO value is linked to an increased risk of ILD development after 2 years of follow-up, while the DLCOΔ and FVCΔ do not predict ILD development.

## 5. Conclusions

We provide the evidence of the reduction of gas exchange as an early sign of lung involvement before any HRCT signs are evident. At earlier time points, our study could not differentiate the predictive value of FVC from DLCO. In the future, further comparisons of FVC and DLCO as predictors will require prospective studies and a careful consideration of potential confounders, such as pulmonary hypertension, cardiac disease, and/or concomitant ILD treatment. The confirmation of a predictive value of decreased DLCO or FVC in SSc patients without confounders may drive the clinician to perform or repeat HRCT to detect ILD as early as possible and to treat it in the patient’s window of opportunity.

## Figures and Tables

**Table 1:** Demographical and clinical characteristics of the enrolled population at t_0_.

Features	Number of Patients (% of Patients with Available Data)
Sex (F)	403 (85.0%)
Cigarette smoking ever	33 (20.8%)
N.A.	315
Current cigarette smoker	14 (26.4%)
N.A.	421
Disease duration (from Raynaud’s phenomenon onset) in months (median; Q1–Q3)	77.4 (28.0–165.8)
Dysphagia	286 (60.5%)
N.A.	1
Renal crisis	6 (1.3%)
N.A.	2
Dyspnoea NYHA stage	
▪ NYHA 1	353 (79.0%)
▪ NYHA 2	89 (19.9%)
▪ NYHA 3	5 (1.1%)
▪ NYHA 4	0
N.A.	27
Palpitations	63 (13.6%)
N.A.	9
Conduction block	50 (11.6%)
N.A.	42
Skin involvement	
▪ dcSSc;	122 (26.0%)
▪ lcSSc;	274 (58.3%)
▪ Only sclerodactyly;	56 (11.9%)
▪ No skin involvement.	18 (3.8%)
N.A.	4
mRSS (mean ± SD)	7.6 ± 7.1
Digital pitting scars	
▪ Current;	36 (26.3%)
▪ Previously;	26 (19.0%)
▪ Never.	75 (54.7%)
N.A.	337
Digital ulcers	
▪ Current;	16 (11.6%)
▪ Previously;	44 (31.9%)
▪ Never.	78 (56.5%)
N.A.	336
Telangiectasia	58 (37.9%)
N.A.	321
SSc-specific Antibody positivity	
▪ ACA;	220 (48.0%) (N.A. 18)
▪ Topo-I;	117 (25.4%) (N.A. 16)
▪ RNA-Pol III.	12 (4.7%) (N.A. 224)
CRP Elevation	50 (10.9%)
N.A.	16
Presence of proteinuria	15 (3.3%)
N.A.	9
Capillaroscopic scleroderma pattern	
▪ Early;	52 (28.3%)
▪ Active;	82 (44.6%)
▪ Late.	50 (27.2%)
N.A.	290

Legend: F: female; N.A.: not available; dcSSc: diffuse cutaneous systemic sclerosis; lcSSc: limited cutaneous systemic sclerosis; mRSS: modified Rodnan skin score; ACA: anticentromere antibodies, Topo-I: anti-topoisomerase I antibodies; RNA-Pol III: anti-RNA polymerase III antibodies.

**Table 2:** Clinical and instrumental characteristics at t_0_ in patients with negative and positive t_2_ HRCT.

Features	Patients with Negative t_2_ HRCT *n* (%)	Patients with Positive t_2_ HRCT *n* (%)	*p*-Value
Gender			
▪ Female;	365 (91.0%)	36 (9.0%)	0.2101
▪ Male.	63 (86.3%)	10 (13.7%)	
Disease duration (from Raynaud onset) in months (median; Q1–Q3) (*n*)	77.8 (28.4–161.7) (206)	71.2 (17.4–194.1) (18)	0.7537
Skin involvement			
▪ dcSSc;	106 (86.9%)	16 (13.1%)	
▪ lcSSc;	250 (91.2%)	24 (8.8%)	
▪ Only sclerodactyly;	52 (92.9%)	4 (7.1%)	0.2832
▪ No skin involvement.	18	0	
mRSS (mean ± SD) (median; Q1-Q3) (*n*)	6 (3–10) (393)	7 (3–13)	0.1563
SSc-specific Antibody positivity			
▪ ACA-positive;	211 (95.9%)	9 (4.1%)	**0.0001**
▪ ACA-negative;	203 (85.3%)	35 (14.7%)	
▪ Topo-I-positive;	97 (82.9%)	20 (17.1%)	**0.0031**
▪ Topo-I-negative;	317 (92.4%)	26 (7.6%)	
▪ RNA-Pol III-positive;	9 (75.0%)	3 (25.0%)	0.1003
▪ RNA-Pol III-negative.	222 (91.0%)	22 (9%)	
PFTs (values reported as %predicted ±SD)			
▪ t_0_ DLCO;	79.0 (±16.6)	69.9 (±17.4)	**0.0006**
▪ t_0_ FVC;	102.2 (±17.3)	94.6 (±16.2)	**0.0052**
▪ t_1_ DLCO;	78.4 (±16.8)	68.9 (±18.6)	**0.0005**
▪ t_1_ FVC;	101.9 (±17.9)	94.7 (±16.5)	**0.0092**
▪ t_2_ DLCO;	78.0 (±17.0)	65.1 (±19.1)	**<0.0001**
▪ t_2_ FVC.	101.6 (±17.6)	94.5 (±20.0)	0.126

Legend: dcSSc: diffuse cutaneous systemic sclerosis; lcSSc: limited cutaneous systemic sclerosis; mRSS: modified Rodnan skin score; ACA: anticentromere antibodies, Topo-I: anti-topoisomerase I antibodies; RNA-Pol III: anti-RNA polymerase III antibodies.

**Table 3:** Percentage of positive-HRCT patients at t_2_ in the populations identified according to the trend in DLCO and FVC from t_0_ to t_1_.

Trend in DLCO from t_0_ to t_1_	Pts with Negative t_2_ HRCT	Pts with Positive t_2_ HRCT	Odds Ratio (Confidence Limits)	*p*-Value
DLCO ≥ 80% at t_0_ and t_1_	152 (94.4%)	9 (5.6%)	Ref	
DLCO ≥ 80% at t_0_ and <80% at t_1_	46 (9.7%)	1 (2.2%)	0.37 (0.05–2.98)	0.3479
DLCO < 80% at t_0_ and ≥80% at t_1_	33 (86.6%)	5 (13.3%)	2.56 (0.81–8.13)	0.1112
DLCO < 80% at t_0_ and at t_1_	197 (86.6%)	31 (13.3%)	2.66 (1.23–5.75)	**0.0130**
**Trend in FVC from t_0_ to t_1_**	**Pts with Negative t_2_ HRCT**	**Pts with Positive t_2_ HRCT**	**Odds Ratio (Confidence Limits)**	***p*-Value**
FVC ≥ 80% at t_0_ and t_1_	380 (91.0%)	35 (8.9%)	Ref	
FVC ≥ 80% at t_0_ and <80% at t_1_	12 (75.5%)	4 (25.5%)	3.62 (1.11–11.82)	**0.0331**
FVC < 80% at t_0_ and ≥80% at t_1_	10 (91.0%)	1 (8.9%)	1.09 (0.14–8.73)	0.9384
FVC < 80% at t_0_ and at t_1_	26 (81.0%)	6 (18.9%)	2.51 (0.97–6.50)	0.0588

## Data Availability

The access to the anonymized raw data that support the findings of this study are available on reasonable request, and subject to review by EUSTAR committee. Data requests may be sent to the corresponding author.

## Abbreviations

SSc	Systemic sclerosis
ILD	Interstitial lung disease
HRCT	High-resolution computed tomography
PFTs	Pulmonary function tests
DLCO	Diffusion of the lung for carbon oxide
FVC	Forced vital capacity
EUSTAR	European Scleroderma Trial and Research
dcSSc	Diffuse cutaneous subset of SSc
lcSSc	Limited cutaneous subset of SSc
ACA	Anticentromere antibodies
Topo-I	Anti-topoisomerase I
RNA-Pol III	Anti-RNA polymerase III antibodies

## Appendix A

List of EUSTAR collaborators:

- Basel (Switzerland) Ulrich Walker, Dr. Bettina Bannert (002);
- Bari (Italy) Florenzo Iannone, Fabio Cacciapaglia fabio.cacciapaglia79@gmail.com (004);
- Genova (Italy) Maurizio Cutolo, Sabrina Paolino sabrina.paolino@unige.it (011);
- Pavia (Italy), Carlomaurizio Montecucco, Roberto Caporali caporali@smatteo.pv.it (019);
- Madrid (Spain), Patricia Carreira, Beatriz E. Joven (023);
- Padova (Italy), Andrea Doria, Elisabetta Zanatta (031);
- Paris (France), Dominique Farge Bancel, Adrian Hij adrian.hij@aphp.fr (035);
- Torino (Italy), Raffaele Pellerito, Torino, Italy (049);
- Tübingen (Germany), Jörg Henes, Ann-Christian Pecher Ann-Christin.Pecher@Med.Uni-Tuebingen.de (056);
- Niska Banja (Serbia and Montenegro), Bojana Stamenkovic, Aleksandra Stankovic ackana@junis.ni.ac.rs (073);
- Moscow (Russia), Lidia P.Ananieva, Ludmila Garzanova lyuda-garzanova@yandex.ru (078);
- Bad Nauheim (Germany), Ulf Müller-Ladner (081);
- Lille (France), David Launay (093);
- Bucharest (Romania), Ruxandra Maria Ionescu, Daniela Opris danaopris0103@yahoo.com (096);
- Bucharest (Romania), Ana Maria Gheorghiu, Mihai Bojinca (100);
- Erlangen (Germany), Jörg Distler (106);
- Milan (Italy); Francesca Ingegnoli (110);
- Gent (Belgium), Vanessa Smith (113);
- Foggia (Italy), Francesco Paolo Cantatore, Addolorata Corrado a.corrado@mail.unifg.it (115);
- Copenhagen (Denmark), Susanne Ullman (116);
- Brussels (Belgium), Marie Vanthuyne, Houssiau frédéric frederic.houssiau@uclouvain.be (122);
- Beijing (China), Mengtao Li (154);
- Alexandria (Egypt), Walid Ahmed Abdel Atty Mohamed (155);
- Roma (Italy), Edoardo Rosato, Antonietta Gigante (158);
- Madrid (Spain), Paloma García de la Peña Lefebvre, Dr. Jorge Juan Gonzalez Martin (169);
- Buenos Aires (Argentina), Eduardo Kerzberg, Fabiana Montoya sfabianamontoya@gmail.com (178);
- Barcelona (Spain), Ivan Castellví, Milena Millan (180);
- New Brunswick, USA, Vivien M. Hsu (188);
- Lubeck (Germany), Gabriela Riemekasten, Sabine Sommerlatte (199).
